# Homœopathy

**Published:** 1880-12

**Authors:** 


THE London *Lancet* thus concludes a reference to the recent Homœopathic Congress at Leeds: "At the dinner in the evening Dr. Yeldham tried to speak comfortably to his brethren on the subject of the slow progress of homœopathy. He thought great reforms were always slow, but considering the reasonableness of the age and the fast rate at which truth and falsehood are exposed, it is certainly becoming a serious argument against homœopathy that eighty years after its promulgation it is as much without scientific recognition as it was two generations back. About the same time that homœopathy was announced Jenner announced the efficacy of vaccination. Let anybody contrast the fate of the two announcements: the one accepted by every civilized country and by every medical school in the world; the other without recognition in any European university, even in Germany, the land of its origin."—*Boston Medical and Surgical Journal.*

